# Synergistic Effects of 25-Hydroxyvitamin D_3_, Phytase, and Probiotics on Growth, Calcium and Phosphorus Metabolism, and Bone Development in Weaned Piglets Fed Low Ca-P Diets

**DOI:** 10.3390/ani16020278

**Published:** 2026-01-16

**Authors:** Baoshi Shi, Saiming Gong, Zhenyang Wang, Jingjing Wang, Cunji Shui, Zhiru Tang, Xie Peng, Yetong Xu, Zhihong Sun

**Affiliations:** 1 Research Center for Bio-Feed and Molecular Nutrition, College of Animal Science and Technology, Southwest University, Chongqing 400715, China; tto93@163.com (B.S.); gsm673628317@email.swu.edu.cn (S.G.); 15660880723@163.com (Z.W.); w20210205111@email.swu.edu.cn (J.W.); shuicunji@email.swu.edu.cn (C.S.); tangzhiru2326@sina.com (Z.T.); pengxie2022@swu.edu.cn (X.P.); 2 Key Laboratory of Chongqing Education Animal Nutrition and Bio-Feed, College of Animal Science and Technology, Southwest University, Chongqing 400715, China

**Keywords:** weaned piglets, 25-OH-VD_3_, phytase, probiotics, calcium and phosphorus metabolism

## Abstract

Seventy 28-day-old weaned barrow piglets (Duroc × Landrace × Large White; 7.2 ± 0.20 kg) were used to determine the effects of 25-hydroxyvitamin D_3_ (25-OH-VD_3_) combined with phytase and probiotics on calcium and phosphorus metabolism and bone development. Five dietary groups were tested: basal diet + 50 µg/kg 25-OH-VD_3_ (CON); basal diet with 17% reduced calcium and phosphorus + 50 µg/kg 25-OH-VD_3_ (LCP); LCP + 50 mg/kg phytase (LH); LCP + 10 mg/kg probiotics (LC); LCP + 50 mg/kg phytase + 10 mg/kg probiotics (LHC). The experiment lasted for 31 days, including 3 days adaptation period. Apparent phosphorus digestibility was higher in the LH and LHC groups than in the CON group (*p* < 0.05). Bone mineral density and calcium content in metacarpal and rib bones were also higher in the LHC group compared with the CON, LCP, LC, and LH groups (*p* < 0.05). The jejunal mRNA expression of solute carrier family 34 members (SLC34A1, SLC34A2, and SLC34A3) members was higher in the LHC group than the CON, LCP, LC, and LH groups (*p* < 0.05), while the relative protein expression of the calcium-sensing receptor in the kidneys was lower in the CON group than in the LCP, LH, LC, and LHC groups (*p* < 0.05). Additionally, supplementation with 25-OH-VD_3_, either alone or in combination with phytase and probiotics, was associated with an increased abundance of beneficial gut bacteria. Overall, combined supplementation of 25-OH-VD_3_, phytase and probiotics enhanced bone development in weaned piglets fed a low-calcium, low-phosphorus diet by improving calcium and phosphorus utilization and calcium–phosphorus metabolic regulation.

## Simple Summary

Optimizing phosphorus utilization in animal feeds is a pivotal factor for sustainable livestock production, yet most current approaches focus on single additives rather than synergistic supplementation strategies. 25-hydroxyvitamin D_3_ (25-OH-VD_3_), phytase, and probiotics exhibit remarkable efficacy in enhancing the absorption and utilization rates of calcium and phosphorus in daily rations. The present study demonstrated that dietary supplementation with 25-OH-VD_3_, administered alone or together with phytase and probiotics, was associated with significant improvements in apparent phosphorus digestibility, calcium and phosphorus metabolism, and bone quality in weaned piglets. Additionally, the inclusion of 25-OH-VD_3_, whether as a standalone additive or in combination with phytase and probiotics, promoted the proliferation of beneficial gut bacteria. Collectively, these findings indicate that the synergistic application of 25-OH-VD_3_, phytase, and probiotics effectively boosts the utilization efficiency of calcium and phosphorus in animal diets.

## 1. Introduction

The weaning phase represents a pivotal juncture in piglet development, coinciding with accelerated bone growth [1]. For optimal skeletal development, piglets require an adequate intake of diverse nutrients. Among these, calcium and phosphorus stand out as indispensable minerals, constituting 65% of the total bone mineral content. Their role in fostering bone growth and maintaining skeletal health is well-documented [2,3]. Insufficient calcium and phosphorus intake in weaned piglets can lead to various skeletal disorders, including stunted growth and rickets [4,5].

Vitamin D_3_ and its metabolite, 25-hydroxyvitamin D_3_ (25-OH-VD_3_), are essential for numerous physiological processes in piglets, especially in the regulation of calcium and phosphorus homeostasis [6,7]. In theory, piglets have the capacity to synthesize vitamin D_3_ from 7-dehydrocholesterol in the skin upon exposure to sunlight [8,9]. Nevertheless, in practical farming scenarios, most piglets are reared in enclosed environments with limited access to sunlight. Consequently, vitamin D_3_ supplementation in the piglets’ diet becomes necessary. Once ingested, vitamin D_3_ is absorbed in the intestine, transported to the liver for conversion into 25-OH-VD_3_, and further metabolized into its most active form, 1,25-dihydroxyvitamin D_3_, in the kidneys [10,11,12,13]. This active metabolite binds to vitamin D receptors on cells, modulating the absorption and utilization of calcium and phosphorus. Maintaining adequate 25-OH-VD_3_ levels is crucial for promoting rapid bone development in piglets. Compared to regular vitamin D_3_, 25-OH-VD_3_ exhibits greater bioactivity, making it a promising alternative feed additive [14,15,16,17].

Phytase, a widely used feed additive, can liberate phosphorus bound by phytic acid in feed, enhancing phosphorus absorption and elevating blood phosphorus levels in piglets [18]. Probiotics, conversely, can modulate the intestinal microbiota and improve gut health [19]. A healthy gut not only facilitates the absorption of fat-soluble vitamins, such as vitamin D, but also stimulates the production of transport proteins, like calcium-binding proteins, enabling piglets to absorb more calcium and phosphorus from the intestine and augment their body stores. Emerging evidence suggests a strong correlation between alterations in the gut microbiome and alterations in bone mass and microstructure [20].

The gut microbiota plays a pivotal role in regulating host calcium homeostasis by enhancing mineral bioavailability and intestinal absorption [21]. Probiotic bacteria can produce enzymes such as phytases, which hydrolyze phytic acid present in plant-derived foods, thereby releasing bioavailable inorganic minerals, including calcium, magnesium, iron, and phosphorus. In addition, probiotics—particularly short-chain fatty acid (SCFA)-producing bacteria—indirectly promote calcium absorption through the fermentation of indigestible polysaccharides [22]. The resulting SCFAs lower gastrointestinal pH, increasing calcium solubility and facilitating its transepithelial transport. Both in vivo and clinical studies have demonstrated positive correlations between the abundance of SCFA-producing gut bacteria, intestinal calcium absorption, and bone mineral density, highlighting a close functional link between probiotic-mediated microbial activity and calcium metabolism [23]. In addition, a growing body of evidence demonstrates the importance of the reciprocal relationship between the gut microbiome and vitamin D. The microbiome and vitamin D mutually regulate each other and jointly modulate host mineral metabolism through multiple mechanisms [24].

The escalating global demand for sustainable livestock production has precipitated a paradigm shift in feed formulation strategies. Contemporary research emphasizes the optimization of phosphorus utilization as a crucial linchpin for sustainable agricultural development [25]. Emerging evidence highlights the critical role of calcium-phosphorus bioavailability in animal growth and environmental stewardship [26]. As demonstrated above, 25-OH-VD_3_, phytase, and probiotics have exceptional efficacy in improving the utilization rates of calcium and phosphorus in daily rations [14,18,20]. Thus, this study aims to evaluate the influence of 25-OH-VD_3_ in combination with phytase and probiotics on skeletal development in weaned piglets fed low calcium-phosphorus diets.

## 2. Materials and Methods

### 2.1. Animal Use, Care, and Ethical Considerations

A total of 70 28-day-old weaned barrow piglets (Duroc × Landrace × Large White; 7.2 ± 0.2 kg) were procured from Dekang Agricultural Technology Development Co., Ltd. (Chongqing, China). Each piglet was housed individually in stainless-steel metabolic cages measuring 1.8 m in length, 1.2 m in width, and 0.80 m in height, ensuring unrestricted access to fresh water throughout the experiment. The ambient temperature in the rearing room was meticulously maintained at 25.4–26.6 °C to provide an optimal environment for the piglets.

### 2.2. Diets and Experimental Design

A total of 70 piglets were randomly assigned to five dietary treatment groups: (1) the control group + 50 µg/kg 25-OH-VD_3_ (CON) received the basal diet; (2) the low calcium and phosphorus + 50 µg/kg 25-OH-VD_3_ group (LCP) received the basal diet with 17% reduced calcium and phosphorus content; (3) the low calcium and phosphorus + phytase + 50 µg/kg 25-OH-VD_3_ group (LH) received the basal diet with 17% reduced calcium and phosphorus, supplemented with 50 mg/kg phytase; (4) the low calcium and phosphorus + probiotic + 50 µg/kg 25-OH-VD_3_ group (LC) received the basal diet with 17% reduced calcium and phosphorus, supplemented with 10 mg/kg probiotics; (5) the low calcium and phosphorus + phytase + probiotic + 50 µg/kg 25-OH-VD_3_ group (LHC) received the basal diet with 17% reduced calcium and phosphorus, supplemented with both 50 mg/kg phytase and 10 mg/kg probiotics. The phytase (product name: Hiphos 2000(GT), additive dosage: 50 g per ton of feed), probiotics (product name: Cylactin, Enterococcus faecium NCIMB10415, additive dosage: 10 g per ton of feed), and 25-OH-VD_3_ (98%, powder) used in this experiment was obtained from DSM (China) Com., Ltd. (Shanghai, China). Each dietary group included 7 replicates, and each replicate comprised 2 piglets. The experiment lasted 31 days, including a 3-day adaptation period at the beginning to acclimate the piglets to the diet and housing conditions. The basal diet was formulated according to the guidelines of the National Research Council [27]. Piglets were fed twice daily at 07:00 and 19:00. The detailed ingredients and nutrient composition of the diets are presented in Table 1.

### 2.3. Recording and Sample Collection

During the experimental period, the daily feed intake of the piglets was meticulously recorded. On days 4 and 32, the piglets were weighed at fasting status in the morning. Subsequently, the average daily body weight gain (ADG), average daily feed intake (ADFI), and feed to gain ratio (F/G) were calculated using the following equations:

ADG = total weight gain/(number of test days × number of replicates);

ADFI = total feed intake/(number of test days × number of replicates);

F/G = Feed/Gain.

Starting from the 25th day of the trial, 0.3% titanium dioxide (TiO_2_) was added to the feed as a digesta marker. Between days 29 and 31, fecal samples (approximately 200 g per pig per collection) were collected. To prevent evaporation and nitrogen loss, each fecal sample was immediately mixed with 10% sulfuric acid with a ratio of 10 mL per 100 g of feces and then stored at −20 °C. Fecal samples from each replicate of every collection period were combined, thoroughly homogenized, and subdivided into subsamples. These subsamples were subsequently dried, ground, and passed through a 1-mm sieve for nutrient analysis.

At the conclusion of the trial, six replicates were randomly selected from each experimental group, and one piglet was randomly chosen from each replicate for slaughter. Prior to slaughter, a 5 mL blood sample was collected from the anterior vena cava of each selected piglet into 10 mL heparinized vacutainer tubes (BD Vacutainer Systems, Franklin Lakes, NJ, USA). The piglets were anesthetized with an intravenous injection of sodium pentobarbital at a dose of 50 mg/kg BW. After euthanasia, the left and right hind limbs were separated. The fascia and muscle tissues were carefully stripped, and following a saline wash, the metatarsals and phalanges of the hind limbs were dissected. These samples were used to determine skeletal indices. The carcasses were then dissected, and the morphology of the main internal organs was observed. The weights of organs including the stomach, heart, liver, spleen, lungs, kidneys and pancreas were measured, and the corresponding organ indices were calculated. Approximately 5 cm of jejunal and colonic tissues were collected as fixed samples and placed in 50 mL centrifuge tubes containing a paraformaldehyde solution to analyse intestinal morphology. Additionally, 10 cm of colon tissue was scraped from the mucosa for the analysis of calcium and phosphorus metabolism and immune-related factors. About 5 cm of colon tissue was collected as molecular samples and stored in freezing tubes, while 10 mL of colonic contents was collected for 16S microbiota analysis. All samples were immediately frozen in liquid nitrogen and subsequently stored at −80 °C.

### 2.4. Chemical Analysis and Calculation

#### 2.4.1. Dietary Compositions

Diet samples were analyzed for dry matter (DM), crude protein (CP), calcium, and total phosphorus. For DM and CP determination, samples were analyzed according to official methods of the Association of Official Analytical Chemists (AOAC) [28]. Specifically, the DM content was measured using AOAC method 930.15, which involves drying the samples to constant weight to remove moisture; the CP content was determined using AOAC method 976.05, based on the Kjeldahl nitrogen determination principle. Calcium and total phosphorus were analyzed in accordance with the National Standard of the People’s Republic of China. The determination of calcium adhered to GB/T 6436-2018 [29], while the total phosphorus content was measured according to GB/T 6437-2018 [30]. The metabolic energy (ME) values of the diets were calculated based on the China Feed Database [31].

#### 2.4.2. Plasma Biochemical Indicators

The biochemical indices in plasma including albumin, globulin, glucose, urea, total cholesterol, calcium, and phosphorus were determined via a colorimetric method. The test kits utilized for this purpose were procured from Jiancheng Technology Co., Ltd. (Nanjing, China). This colorimetric approach relies on the specific chemical reactions of each analyte in the plasma samples with the reagents in the kits, which generate color products proportional to the concentration of the target substances. The absorbance of these colored products is then measured, and plasma biochemical indices are calculated from pre-established standard curves.

For the detection of 1,25-(OH)2-D_3_, 25-OH-VD_3_, anti-tartrate acid phosphatase, bone Alkaline phosphatase, parathyroid hormone, calcitonin, an enzyme-linked immunosorbent assay (ELISA) was employed. The ELISA kits were sourced from Shanghai Enzyme-linked Biotechnology Co., Ltd. (Shanghai, China). This highly sensitive and specific immunoassay technique uses antibodies that bind target immunoglobulins and cytokines. Through a series of incubation, washing, and substrate-enzyme reactions, the bound analytes are detected, and their concentrations in the plasma samples are quantified according to the calibration curves made by the standard substances in the reagent the kits.

#### 2.4.3. Intestinal Mucosa Morphology

Hematoxylin-eosin staining was performed for the histological examination of fixed jejunal tissue samples. The procedure consisted of the following steps: sampling, ethanol dehydration, xylene transparency, paraffin embedding, sectioning, staining, microscopic observation, and determination of villi height, crypt depth, and the number of cup cells.

#### 2.4.4. Quantitative PCR Analysis

The expression levels of target mRNA in renal and colonic mucosal tissues were analyzed by real-time quantitative PCR. Oligonucleotide primers were designed with Primer Premier 5 software, validated through the NCBI GenBank database, and commercially synthesized by Shenggong Biotech (Shanghai, China). The specific primer sequences are provided in Table S1 [26]. Tissue RNA isolation was performed using a total RNA extraction kit (Accurate Biology, Changsha, China) according to the manufacturer’s protocol. For qPCR analysis, a fluorescence quantitative PCR kit (Tianjing, China) was employed with the following 20 μL reaction system: 10 μL 2 × ChamQ SYBR qPCR Master Mix, 0.4 μL each of forward and reverse primers (10 μM), 0.4 μL 50 × ROX Reference Dye 1, 2 μL cDNA template, and 6.8 μL nuclease-free water. The cycling conditions were as follows: 95 °C enzyme activation for 2 min, followed by 40 cycles of 95 °C for 15 s, 60 °C for 60 s, and 60 °C for 10 s. The relative quantitative expression difference was calculated using the 2^−∆∆Ct^ method, where ∆∆Ct = (Ct_target gene_ − Ct_β-actin_) experimental group − (Ct_target gene_ − Ct_β-actin_) control group.

#### 2.4.5. Skeletal Performance Measurement

#####  Determination of Defatted Bone Weight

The determination of defatted bone weight was performed based on the procedures described by Wensley et al. [32]. Briefly, the separated ribs, phalanges and metatarsals were subjected to a series of treatments, including steaming and boiling to remove adherent muscle and connective tissue, followed by drying. Subsequently, these bone samples were immersed in an ethanol: benzene mixture (2:1) for 96 h to achieve complete degreasing. After the degreasing process, the samples were taken out and placed in an oven preheated to 105 °C. They were baked until the ethanol-benzene mixture was completely volatilized and a constant weight was obtained. Finally, the weight of the degenerated bone was accurately measured.

##### Determination of Bone Mineral Density

Bone mineral density was assessed using the procedures reported by Keenan et al. [33,34]. All measurements were conducted at 20 °C. First, using an analytical balance, the ribs, metatarsal and toe bones of the previously degreased samples were weighed, and the masses were recorded as M. Subsequently, a 250 mL graduated cylinder was filled with distilled water using a wash bottle to the 10 mL mark. The volume of the distilled water at this point, denoted as V1, and the combined mass of the cylinder and distilled water, recorded as M1, were carefully noted. The pre-weighed metatarsal bones and toe bones were gently placed into the cylinder. Additional distilled water was then added with the wash bottle until the lowest measurable volume on the scale was reached. The new volume of the liquid in the cylinder was recorded as V2, and the combined mass of the cylinder, distilled water, and the bones was noted as M2. Then, the cylinder was refilled with distilled water to the minimum measurable volume, ensuring consistent conditions for accurate measurement. The mass of the additional distilled water added due to the presence of the bones, M3, was calculated as M3 = M2 − M1 − M. According to Archimedes’ principle, where volume = mass/density, the additional volume of distilled water, V3, was calculated as V3 = M3 divided by the density of distilled water at 20 °C (the density of distilled water at 20 °C is 0.99809 g/cm^3^). The volume of the bone tissue, V_bone, was then determined as V_bone = V2 − V1 − V3. Finally, the bone tissue density was calculated by dividing the bone mass, M_bone (equal to M), by the bone volume, V_bone, yielding the bone density.

##### Determination of Bone Ash, Calcium and Phosphorus

The bones for which the defatted bone weight and bone density had been previously measured were placed into crucibles. These crucibles were then ashed at 600 °C for 16 h until the bones turned completely white and reached a constant weight. After cooling, the ash weight was measured, and the ash content was calculated from the initial bone sample weight [35]. For calcium determination, the bone ash was dissolved in nitric acid, and phosphate ions were selectively precipitated as bismuth (III) phosphate (BiPO_4_). The calcium content in the resulting solution was then quantified by a complexometric titration method using disodium ethylenediaminetetraacetic acid (EDTA, disodium versenate) as the titrant. The titration was performed in the presence of a mixed indicator consisting of calcein and thymolphthalein, and calcium concentration was calculated based on the volume of EDTA consumed. Bone phosphorus content was determined using the molybdenum blue colorimetric method [36]. Briefly, 1 mL of the sample solution was transferred into a 50 mL tube, followed by the addition of 2 mL ammonium molybdate solution and 1 mL ascorbic acid, which served as the chromogenic reagent and deoxidizer, respectively. After color development, the absorbance was measured using a spectrophotometer at 710 nm. Phosphorus concentration was quantified using a phosphate standard curve ranging from 1 μg/mL to 6 μg/mL, and the phosphorus content of the bone samples was calculated accordingly.

#### 2.4.6. Western Blotting

The total protein extracted from the renal tissues was used for Western blot analysis. Equal amounts of protein (20 μg per sample) were separated by sodium dodecyl sulfate–polyacrylamide gel electrophoresis (SDS–PAGE) and electrophoresed alongside a pre-stained protein ladder (Bio-Rad, Hercules, CA, USA). The separated proteins were then electrotransferred onto polyvinylidene fluoride (PVDF) membranes. After transfer, the membranes were blocked with 5% (*w*/*v*) bovine serum albumin for 1 h at room temperature and then incubated overnight at 4 °C with the primary antibodies, including Anti-β-actin antibody (8115-1-RR, Proteintech, Rosemont, IL, USA, diluted 1:5000), Anti-CYP27B1 antibody (PA5-79128, Thermo Fisher Scientific, Waltham, MA, USA, diluted 1:2000), and Anti-CASR antibody (19125-1-AP, Proteintech, diluted 1:1000). Following three washes with TBST, the membranes were incubated with a rabbit recombinant secondary antibody (RGAR001, Proteintech) diluted 1:5000 at room temperature. Protein bands were visualized by using enhanced chemiluminescence, and β-actin was used as the internal loading control.

#### 2.4.7. Intestinal Microbiota

Analysis of the microbial composition of the colon was performed using 16S rRNA gene sequencing techniques. Total genomic DNA was extracted from colonic content samples using a commercial DNA extraction kit. The V3–V4 hypervariable regions of the bacterial 16S rRNA gene were amplified by PCR and the PCR products were purified, quantified, and pooled in equimolar concentrations. Sequencing libraries were constructed and subjected to paired-end sequencing on the Illumina MiSeq/NovaSeq platform (Meiji Biotechnology Co., Ltd., Shanghai, China).

Raw sequencing reads were merged and quality-filtered to remove low-quality sequences and chimeras. High-quality sequences were then clustered into operational taxonomic units (OTUs) at 97% sequence similarity (or denoised to generate amplicon sequence variants, ASVs, if applicable). Taxonomic classification was performed by comparing representative sequences against a reference database. The relative abundance of each taxon was calculated to characterize microbial community composition.

Alpha diversity including the Shannon and Simpson indices, were calculated to assess microbial diversity within samples. Beta diversity was evaluated using principal coordinate analysis (PCoA) and non-metric multidimensional scaling (NMDS) based on distance matrices to compare differences in microbial community structure among groups. Differentially abundant taxa between groups were analysed and correlation analyses were conducted to evaluate associations between microbial taxa and experimental variables. Functional profiles of the microbial communities were predicted based on 16S rRNA gene data to infer potential metabolic pathways.

### 2.5. Statistical Analysis

Experimental data were compiled and analyzed using Excel 2023. Statistical analyses were conducted with SPSS 27.0.1, employing the following mixed-effects model: Y_ij_ = µ + T_i_ + S_j_ + ɛ_ij_. In this equation, Y_ij_ represents the observation of the dependent variable, µ the overall mean, T_i_ the fixed effect of treatment, S_j_ the random pig effect, and ɛ_ij_ the residual error for the observation. Duncan’s multiple range test was applied for multiple comparisons, and results are presented as the mean ± standard error of the mean (SEM). Statistical significance was defined as *p* < 0.05. Partial least squares path modeling (PLS-PM) was conducted using R 4.4.1, with the *dplyr* 1.1.4 package employed for data processing and transformation. A goodness-of-fit value exceeding 0.6 is considered excellent.

## 3. Results

### 3.1. Growth Performance and Nutrient Apparent Digestibility

As shown in Table 2, growth performance and apparent nutrient digestibility of pigs did not differ among treatments, with no significant differences in body weight gain (BWG), ADG, ADFI, or F/G (*p* > 0.05). There were no differences in the apparent digestibility of DM and calcium. There was a significant difference in the CP apparent digestibility among the five groups of piglets (*p* = 0.007). Specifically, the parameter for piglets in the CON group was significantly higher than that of piglets in the LC, LHC, and LCP groups, with no difference observed between the LH and LCP groups. The phosphorus level parameter shows a significant difference among the five groups (*p* < 0.005). The CON group is significantly less than the LH and LHC groups (*p* < 0.05). The LCP and LC groups have higher values than the CON group (*p* < 0.05).

### 3.2. Organ Index

As shown in Table 3, no significant differences were observed among the five groups in the organ indices of the heart, liver, spleen, lung, kidney, pancreas, or stomach at the end of the experiment (*p* > 0.05).

### 3.3. Serum Biochemical Indices

As shown in Table 4, there was no difference in the serum albumin, calcium, 1,25-(OH)2-D_3_, 25-OH-VD_3_, anti-tartrate acid phosphatase, and calcitonin content of piglets among the five groups (*p* > 0.05). Dietary treatments caused significant difference in the serum total cholesterol content of piglets (*p* < 0.001), specifically, the parameter of piglets in the CON group was lower than that of piglets in the LCP, LH, LC, and LHC groups (*p* < 0.05), the parameter of piglets in the LCP group was higher than that of piglets in the LH, LC, and LHC groups (*p* < 0.001). The LHC diet significantly increased serum bone alkaline phosphatase activity in weaned piglets compared to the CON, LCP, LHC, and LC diets (*p* < 0.05). There was a significant difference between LCP and LH in the serum parathyroid hormone levels (*p* < 0.05). Dietary treatments caused a significant difference in the serum glucose content of piglets (*p* < 0.05), specifically, the parameter of piglets in the CON group was higher than that of piglets in the LCP, LH, LC, and LHC groups (*p* < 0.05).

### 3.4. Bone Mass

As indicated in Table 5, there was a significant difference in the metacarpal bone mineral density of piglets among five groups (*p* < 0.001), specifically, the parameter of piglets in the CON group and LC group was lower than that of pigs in the LCP, LH, and LHC groups (*p* < 0.001) and the parameter of piglets in the LCP group and LH group was lower than that of piglets in the LHC groups (*p* < 0.001). The calcium and phosphorus content and skimmed bone weight in the metacarpal bone were not different among weaned piglets in the five groups (*p* > 0.05). Dietary treatments resulted in a significant difference in rib bone mineral density among the five groups (*p* < 0.05). Specifically, the parameter of piglets in the LHC group was higher than that of pigs in the CON, LCP, LH, and LC groups (*p* < 0.05). The skimmed bone weight in the rib bones was significantly higher in weaned piglets in the LHC groups than in the CON, LCP, LH, and LC groups (*p* < 0.05). For the calcium and phosphorus levels in the rib bones, there was no difference among the five groups (*p* > 0.05).

### 3.5. Intestinal Mucosal Morphology

**Figure 1:**
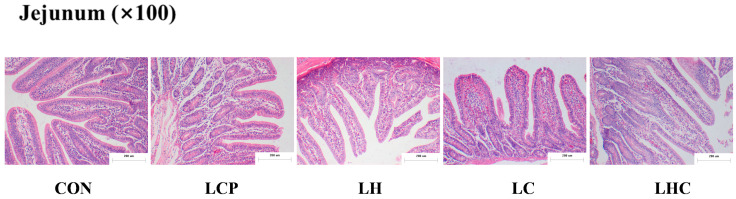
The morphology of jejunal mucosa. Data are presented as means (*n* = 7).

As shown in Table 6, there was a significant difference in the jejunal villus height of piglets among five groups (*p* = 0.001), specifically, the parameter of piglets in the CON group was significantly higher than that of piglets in the LCP, LH, and LC groups (*p* < 0.001) and the parameter of piglets in the LCP groups was significantly lower than that of piglets in the other group (*p* < 0.001). The piglets in the CON group had a higher jejunal crypt depth than those in the LCP, LH, LC, and LCH groups (*p* < 0.001). There was significant difference in the ratio of jejunal villus height to crypt depth (V/C) of piglets (*p* < 0.001), specifically, the parameter of piglets in the LCP groups was significantly lower than that of piglets in the CON, LH, LC, and LHC groups (*p* < 0.001) and the parameter of piglets in the CON and LH groups was lower than that of piglets in the LC and LHC groups (*p* < 0.05). As shown in Figure 1, piglets in the CON group had slight damage and absence of the tips of the villi of the jejunum, while piglets in the LCP group had villi of the jejunum that were not as long as the others. However, the tips of the villi of the jejunum of the weaned piglets in the LC and LHC groups had better morphology, with no obvious fragmentation or absence, and with complete outlines.

### 3.6. mRNA Expression of Calcium and Phosphorus Metabolism Genes in the Kidney and Jejunal Mucosa

**Figure 2:**
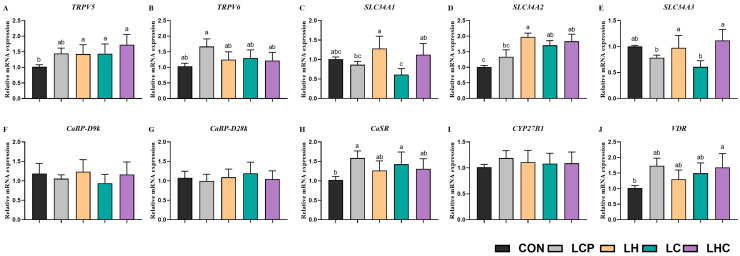
Effects of 25-OH-VD_3_ combined with phytase and probiotics on the jejunal mRNA expression for calcium and phosphorus metabolism in weaned piglets. ^a, b, c^ Values within a row with different superscripts differ significantly (*p* < 0.05; *n* = 6). Abbreviations: TRPV5/TRPV6, transient receptor potential cation channel subfamily V member 5/member 6; SLC34A1/SLC34A2/SLC34A3, solute carrier family 34 (type II sodium/phosphate transporter), member 1/member 2/member 3; CaBP-D9k, calcium-binding protein D9k; CaBP-D28k, calcium-binding protein D28k; CYP27B1, cytochrome P450 27B1; VDR, vitamin D receptor; CaSR, calcium sensing receptor. Same as the figures below.

Figure 2 shows the jejunal relative mRNA expression levels of calcium and phosphorus metabolism genes in weaned piglets. There was an increase of the relative mRNA expression level of *TRPV5* in the jejunum mucosa of weaned piglets in the LHC group than in the CON, LCP, LH, and LC group (*p* < 0.05). Additionally, the relative mRNA expression levels of *CaSR*, *TRPV6*, and *VDR* were significantly higher in the jejunum mucosa of weaned piglets in the LCP group than in the CON, LH, LC, and LHC groups (*p* < 0.05). The jejunum relative mRNA expression levels of *SLC34A1*, *SLC34A2*, and *SLC34A3* in the LH and LHC groups were significantly higher than those in the CON, LCP, and LC groups (*p* < 0.05). The results showed that the dietary treatment did not affect the relative mRNA expression levels of jejunal *CaBP-D9K*, *CaBP-D28*, *CYP27B1* in weaned piglets (*p* > 0.05).

**Figure 3:**
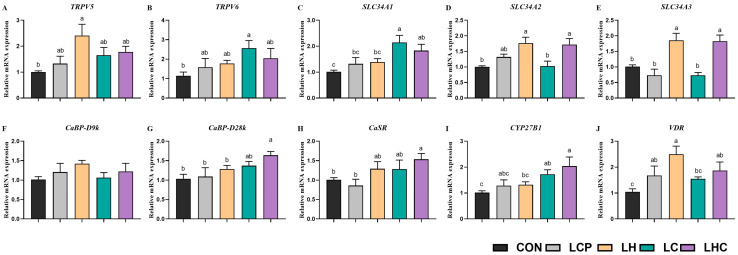
Effects of 25-OH-VD_3_ combined with phytase and probiotics on mRNA expression for calcium and phosphorus metabolism in the kidney of piglets. ^a, b, c^ Values within a row with different superscripts differ significantly (*p* < 0.05; *n* = 6).

Figure 3 shows the relative mRNA expression levels of calcium and phosphorus metabolism genes in the kidneys of weaned piglets. When compared with piglets in the CON group, piglets in the LH group showed higher relative mRNA expression levels of *TRPV5* and *VDR* in the kidney (*p* < 0.05); piglets in the LC group were higher relative mRNA expression levels of *TRPV6* and *SLC34A1* in the kidney (*p* < 0.05); piglets in the LHC group were higher relative mRNA expression levels of *CaBP-28K* and *CASR* in the kidney (*p* < 0.05); piglets in both LH and LHC group were higher relative mRNA expression levels of *SLC34A2* and *SLC34A3* in kidney. Dietary treatments did not significantly affect the relative mRNA expression of *CaBP-D9K* in piglet kidneys (*p* > 0.05).

### 3.7. Protein Expression Related to Calcium and Phosphorus Metabolism in the Kidney

**Figure 4:**
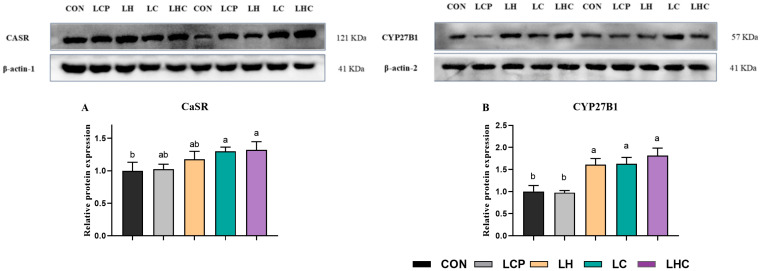
Effects of 25-OH-VD_3_ combined with phytase and probiotics on kidney protein expression for calcium and phosphorus metabolism in weaned piglets. ^a, b^ Values within a row with different superscripts differ significantly (*p* < 0.05; *n* = 4).

Figure 4 shows the kidney protein expression levels of calcium and phosphorus metabolism in weaned piglets. The relative protein expression of CaSR in the kidneys of piglets in the CON group was lower than that in the LC, and LHC groups (*p* < 0.05). The dietary treatments caused a significant difference in the relative protein expression of CYP27B1 in the kidneys of piglets among five groups (*p* < 0.001), specifically, the parameter of piglets in the CON groups was lower than that of piglets in the LH, LC, and LHC groups and the no significant differences were observed among LCP, LH and LHC groups.

### 3.8. Colon Microbiota

**Figure 5:**
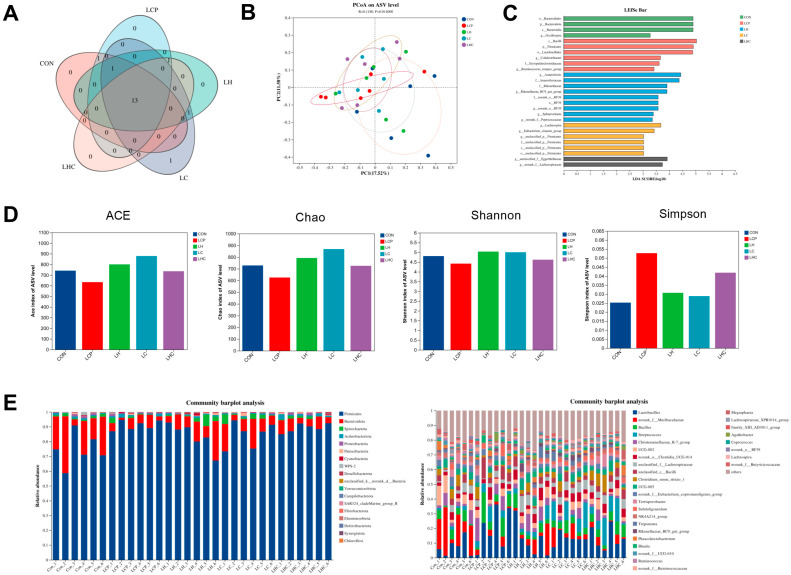
Effects of 25-OH-VD_3_ combined with phytase and probiotics on the colonic microbiota of weaned piglets. (**A**) Venn diagram showing shared and unique ASVs among the five groups. (**B**) PCoA analysis of microbial community similarities. (**C**) LEfSe analysis identifying differentially abundant biomarkers. (**D**) Alpha diversity indices (Chao1, ACE, Shannon, and Simpson). (**E**) Taxonomic composition of the colonic microbiota. ASV, amplicon sequence variant; PCoA, principal coordinate analysis. *n* = 5.

The distributions of shared and specific amplicon sequence variants (ASVs) among the five groups are shown in Figure 5A. The number of shared ASVs among the five groups was 166. The number of specific ASVs in the CON group was 15, that in the LCP group was 11, that in the LH group was 8, the number of specific ASVs in the LC group was 2, and that in the LHC group was 5. In this study, the PCoA results showed the LCP, LH, LC, and LCH groups had similar internal distances and species compositions, whereas the CON group formed different clusters from the LCP and LC groups (Figure 5B). Additionally, the LHC and LC groups exhibited greater internal distances and relatively higher species diversities. Left-side analysis (LDA > 2), as shown in Figure 5C, revealed that the colonic microbiota of weaned piglets in the CON group was significantly enriched in *Bacteroidales*, *Bacteroidota*, *Bacteroidia*, and *Oscillospira*. The colonic microbiota of weaned piglets in the LCP group was significantly enriched in the *Bacilli*, *Firmicutes*, *Lactobacillales*, *Colidextribacter*, *Erysipelatoclostridiaceae*, and *Ruminococcus* groups. The colonic microbiota of weaned piglets in the LH group was significantly enriched in the *Anaerofustis*, *Anaerofustaceae*, *Rikenellaceae*, *Sphaerochaeta*, and *Peptococcaceae*. And *Lachnospira* and *Eubacterium* were significantly enriched in the colonic microbiota of weaned piglets in the LC group compared to the CON, LCP, LH, and LHC groups; *Eggerthellaceae* and *Lachnospiraceae* were significantly enriched in the colonic microbiota of weaned piglets in the LHC group compared to the CON, LCP, LH, and LC groups. Alpha diversity is shown in Figure 5D, there were no significant differences in the colonic microbial ACE, Chao1, Shannon, and Simpson indices among the groups (*p* > 0.05).

The taxonomic composition of the colonic microbiota in weaned piglets is presented in Figure 5E. At the phylum level, the ten most abundant phyla across the five treatment groups included Firmicutes, Bacteroidota, and Spirochaetota. Firmicutes and Bacteroidota predominated in the colonic microbiota of all groups, followed by Spirochaetota. At the genus level, *Lactobacillus*, *Muribaculaceae*, *Bacillus*, and *Streptococcus* were the dominant genera in the colonic microbiota of weaned piglets. The relative abundance of *Lactobacillus* in the LCP group was significantly higher than the CON, LH, LC, and LCH groups (*p* = 0.004). Conversely, the abundance of *Muribaculaceae* was significantly greater in the CON group than in the LCP, LH, LC, and LCH groups (*p* < 0.05). In addition, *Streptococcus* abundance in the LC and LCH groups was significantly higher than the CON, LCP, and LH groups (*p* < 0.05).

### 3.9. Correlation Analysis

**Figure 6:**
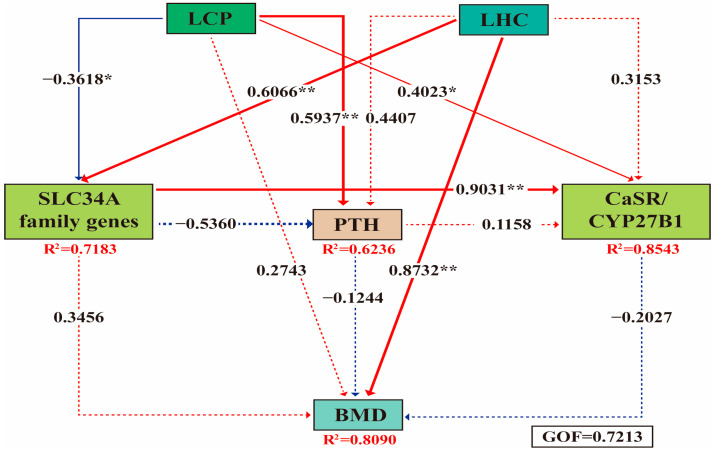
Correlation analysis of the regulatory mechanism of 25-OH-VD_3_ combined with phytase and probiotics on bone development in weaned piglets using PLS-PM. Solid lines indicate statistically significant differences, while dashed lines represent non-significant differences. Red and blue colors denote positive and negative correlations, respectively. * indicates 0.01 < *p* < 0.05, and ** denotes *p* < 0.01. Abbreviations: GOF, goodness-of-fit; PTH, parathyroid hormone.

Results from the PLS-PM analysis revealed that the LCP group primarily promoted the release of calcium and phosphorus from bones into the blood through parathyroid hormone (PTH) (*β* = 0.594, *p* < 0.01), thereby maintaining normal blood calcium and phosphorus levels. The LHC group primarily enhanced calcium and phosphorus absorption by intestinal epithelial cells by upregulating SLC34A family genes (*β* = 0.607, *p* < 0.01), thereby increasing blood calcium and phosphorus levels. Meanwhile, 25-OH-VD_3_ synergistically contributed to maintaining blood calcium and phosphorus levels, and further upregulated the CYP27B1 gene in the kidneys (*β* = 0.402, *p* < 0.05), thereby promoting the activation of 25-OH-VD_3_. High blood calcium and phosphorus levels triggered a negative feedback mechanism, inducing the expression of the CaSR gene in the kidneys to maintain homeostasis of blood calcium and phosphorus (Figure 6).

## 4. Discussion

### 4.1. Growth Performance and Organ Development

In this study, the impact of adding 25-OH-VD_3_, along with low calcium and phosphorus, either alone or in combination with phytase (Hiphos), probiotics (Cylactin), or a combination of phytase and probiotics, to piglet diets was investigated. These additives did not cause significant differences in piglet growth performance, including BWG, ADG, ADFI, and F/G. This is consistent with the previous report, which found that dietary inclusion of phytase or 25-OH-VD_3_ had no effect on ADG, final carcass weight, kill-out percentage, back fat depth, muscle depth, or lean meat percentage in finishing pigs [37]. Currently, knowledge regarding the effects of 25-OH-VD_3_ on the performance of weaned piglets remains limited.

However, this study revealed that adding 25-OH-VD_3_ to low-calcium, low-phosphorus diets, or in combination with phytase, probiotics, or both, increased the apparent phosphorus digestibility in piglets. Similar findings have been reported previously, where finishing pigs fed diets supplemented with 25-OH-VD_3_ showed improved apparent digestibility of phosphorus and ash [37], and laying hens with dietary supplementation of different forms of VD_3_ had enhanced apparent digestibility of calcium and phosphorus [38]. Phytase, a commonly used feed additive that releases phosphorus bound by phytic acid, has been widely studied in pig feeding [37,39,40]. The current study also showed that adding 25-OH-VD_3_ alone or in combination with phytase to the diet improved the apparent digestibility of calcium in weaned piglets.

Furthermore, no significant differences were observed in the organ indices, including the heart, liver, spleen, lung, kidney, pancreas, and stomach, among the different dietary treatments. Specifically, dietary supplementation with 25-OH-VD_3_, either alone or in combination with phytase and probiotics, did not significantly affect the renal index of weaned piglets. As a key metabolic organ involved in calcium and phosphorus homeostasis and detoxification, the kidney is highly sensitive to mineral imbalance [41,42]. The absence of significant changes in renal index suggests that the combined supplementation strategies did not induce adverse effects on renal development or function under low calcium and phosphorus conditions. Overall, these results indicate that dietary inclusion of 25-OH-VD_3_, phytase, and probiotics is safe with respect to major organ development in weaned piglets.

### 4.2. Effects on Serum Biochemical Indices

Serum calcium and phosphorus levels can reflect skeletal calcium and phosphorus metabolism. In this study, adding 25-OH-VD_3_ to both normal- and low-calcium, low-phosphorus diets did not result in different serum calcium and phosphorus levels among the five groups. This finding is consistent with a previous report that adding 25-OH-VD_3_ to sow diets had no significant effect on blood calcium or phosphorus levels [43]. The tendency toward a lower serum phosphorus concentration observed in the LC group may be attributed to the effects of probiotic supplementation under low calcium and phosphorus conditions. Probiotics are known to improve intestinal function and nutrient utilization efficiency, which may facilitate phosphorus uptake and its subsequent incorporation into tissues rather than increasing circulating phosphorus levels. In addition, the absence of phytase in the LC diet may have limited the hydrolysis of phytate-bound phosphorus, thereby restricting phosphorus availability in the circulation. These findings suggest that probiotic supplementation alone may modulate phosphorus metabolism by altering its intestinal handling and tissue distribution, whereas the combined supplementation of probiotics and phytase appears to be more effective in maintaining serum phosphorus homeostasis.

In addition to mineral-related indices, several metabolic and endocrine parameters were also affected by dietary treatments. Serum glucose concentration differed significantly among groups, with piglets in the LCP, LC, and LHC groups exhibiting lower glucose levels compared with the CON group. This reduction in circulating glucose may reflect improved metabolic efficiency and reduced metabolic stress under low calcium and phosphorus conditions when diets were supplemented with functional additives. Previous studies have suggested that phytase and probiotics can enhance nutrient digestibility and energy utilization, thereby reducing the reliance on circulating glucose as a rapid energy source [44]. The significantly lower serum urea nitrogen concentration observed in the LHC group indicates an improvement in nitrogen utilization efficiency. The combined supplementation of phytase and probiotics under low Ca and P conditions likely enhanced mineral availability, protein digestibility, and intestinal function, thereby reducing amino acid catabolism and promoting protein retention. Moreover, the presence of 25-OH-VD_3_ may have further optimized Ca–P homeostasis and protein metabolism, resulting in decreased urea formation. Cholesterol plays a crucial role in metabolism, as it can be converted into vitamin D when the skin is exposed to sunlight, which is essential for calcium absorption and maintaining bone health. In this study, the addition of both phytase and probiotics increased total serum cholesterol on a low-calcium, low-phosphorus diet, which is inconsistent with a previous report that found adding phytase to broiler diets decreased serum total cholesterol [45].

Bone alkaline phosphatase (BALP), a marker of osteoblastic activity and bone formation, was significantly affected by dietary treatment. The LHC group exhibited a markedly higher BALP concentration compared with the other groups, indicating enhanced bone formation activity when phytase and probiotics were combined with 25-OH-VD_3_ under low Ca and P conditions. This increase in BALP corresponded with more favorable mineral and hormonal profiles, supporting improved skeletal metabolism rather than merely altered serum mineral concentrations. Parathyroid hormone (PTH), secreted by the parathyroid glands, plays a crucial role in regulating calcium and phosphate homeostasis. In this study, the addition of probiotics increased PTH serum levels on a low-calcium, low-phosphorus diet, consistent with a previous report showing that probiotics can interact with PTH through the effects of gut microbiota metabolites [46].

### 4.3. Calcium and Phosphorus Metabolism

Calcium and phosphorus, which exist in bones as both amorphous calcium phosphate and crystalline hydroxyapatite [47], play a crucial role in maintaining physiological functions. Under conditions of inadequate calcium intake or impaired absorption, calcium is mobilized from bone to maintain circulating calcium homeostasis, as demonstrated in several studies [47,48,49,50]. Kaastad et al. [51] demonstrated that a low-calcium diet could induce osteoporosis in rats, as evidenced by decreased bone calcium content, underscoring the importance of bone calcium and phosphorus levels as indicators of skeletal mineral metabolism. In this study, adding 25-OH-VD_3_ to the diet increased the bone mineral density in metatarsals of weaned piglets. Moreover, combining 25-OH-VD_3_ with phytase and probiotics increased bone mineral density and calcium content in metacarpals and ribs. In contrast, no significant differences were observed in the bone density and calcium-phosphorus content of metacarpals and ribs among the CON, LCP, LH, and LC groups, which diverged from previous studies. For instance, Regassa et al. [5] reported that supplementing the diet of growing pigs with 25-OH-VD_3_ did not affect their bone mineralization parameters, indicating that adding 25-OH-VD_3_ alone or in combination with phytase and probiotics could enhance the bone mineral density of weaned piglets.

Calcium and phosphorus absorption and metabolism in animals primarily take place in the intestine and kidney. Notably, the current study found that dietary supplementation with 25-OH-VD_3_ increased mRNA or protein expression of relevant genes. For example, it upregulated the relative mRNA levels of *TRPV5*, *CASR*, *CaBP-D28K*, *SLC34A2, CYP27B1*, and *VDR*, as well as *CASR* protein expression in the kidney. On the basis of adding 25-OH-VD_3_, supplementing the diet with phytase, or combining phytase with probiotics further increased mRNA and protein expression of certain genes to varying degrees. In bone metabolism, both bone formation and resorption depend on the transcellular transport of calcium ions mediated by the TRPV5 and TRPV6 channels. Calcium ions enter cells through TRPV5 and TRPV6, bind to CaBP-28K and CaBP-9K, and subsequently diffuse toward the basolateral membrane, where they are extruded by calcium pumps [52,53]. Increased expression of TRPV5 and TRPV6 may reflect elevated intracellular calcium concentrations and enhanced calcium influx, thereby facilitating cellular adaptation to physiological stimuli [54]. In addition, CASR, a member of the C family of G protein–coupled receptors, is widely expressed in the gastrointestinal tract, kidneys, and bone tissue and plays a crucial role in the regulation of calcium homeostasis [6,55]. Predominantly expressed in the parathyroid glands and kidneys, CASR functions as a key regulator of renal calcium handling in response to fluctuations in circulating calcium levels [56,57], thereby contributing to the maintenance of systemic calcium balance and bone homeostasis. Although Regassa et al. [5] found that supplementing diets with 50 and 100 mg 25-OH-VD_3_ kg^−1^ increased the relative mRNA expression level of *SLC34A1* in the duodenum of growing pigs without affecting *TRPV6* relative mRNA expression levels, our results suggest that adding 25-OH-VD_3_ alone or in combination with phytase or probiotics can improve cellular calcium and phosphorus absorption and utilization.

### 4.4. Gut Microbiota

The gut microbiota comprises hundreds of millions of microorganisms, including commensal, pathogenic, and neutral bacteria, which collectively contribute to maintaining intestinal homeostasis [58]. In the present study, dietary supplementation with 25-OH-VD_3_ combined with probiotics increased the abundance of Eubacterium. Moreover, supplementation with 25-OH-VD_3_ in combination with both phytase and probiotics increased the relative abundance of Eggerthellaceae and Lachnospiraceae at both the family and genus levels in the colonic microbiota of weaned piglets. The abundance of Lactobacillus in the colonic microbiota of piglets in the LCP group was significantly higher than that in the CON, LH, LC, and LCH groups. In contrast, the abundance of Muribaculaceae was significantly higher in the CON group than in the LCP, LH, LC, and LCH groups. Additionally, the abundance of Streptococcus in the LC and LCH groups was significantly greater than that in the CON, LCP, and LH groups. To date, information regarding the effects of dietary supplementation with 25-OH-VD_3_ on gut microbiota composition remains limited. The findings of this study suggest that supplementation with 25-OH-VD_3_, particularly when combined with phytase or probiotics, exerts beneficial effects on the intestinal microbiota of weaned piglets.

Probiotics enhance intestinal Ca^2+^ absorption and bone health through multiple and complementary mechanisms [59]. A healthy gut microbiota improves Ca^2+^ bioaccessibility and regulates gut-derived serotonin, which indirectly modulates bone metabolism via osteoblast and osteoclast activity [60,61]. Probiotic supplementation, particularly with Lactobacillus strains, has been shown to enhance both transcellular and paracellular Ca^2+^ transport by upregulating vitamin D receptors, Ca^2+^ transporters, and tight junction proteins [62]. Collectively, evidence from in vitro studies, animal models, and randomized clinical trials demonstrates that probiotic supplementation improves Ca^2+^ absorption, restores Ca balance, and increases bone mineral density.

### 4.5. Mechanism of Action

**Figure 7:**
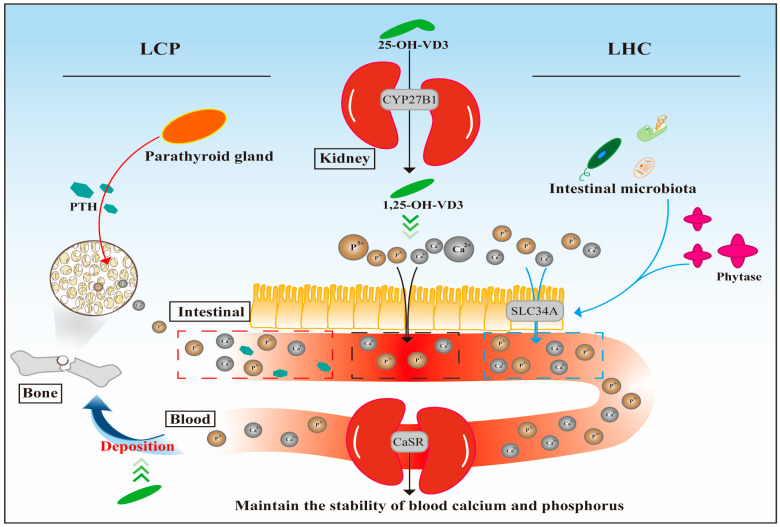
Visualization of the regulatory mechanism of 25-OH-VD_3_ combined with phytase and probiotics on bone development in weaned piglets. Abbreviations: CYP27B1, cytochrome P450 27B1; LCP, basal diet with 17% reduced calcium and phosphorus + 50 µg/kg 25-OH-VD_3_; LHC, basal diet with 17% reduced calcium and phosphorus + 50 mg/kg phytase + 10 mg/kg probiotics + 50 µg/kg 25-OH-VD_3_.

PLS-PM analysis revealed that the LCP group primarily increases blood calcium and phosphorus levels through the body’s feedback regulation, whereas the LHC group mainly raises these levels by promoting their intestinal absorption. The elevation of blood calcium and phosphorus further triggers a feedback regulatory mechanism: the expression of the CaSR gene in the kidneys promotes the excretion of these ions, thereby maintaining their stability. Meanwhile, 25-OH-VD_3_ is converted to 1,25-OH-VD_3_ by the CYP27B1 enzyme in the kidneys, which not only promotes the absorption of calcium and phosphorus but also facilitates their deposition in bones, thereby maintaining bone density (Figure 7).

## 5. Conclusions

In summary, dietary supplementation with 25-OH-VD_3_ in combination with phytase and probiotics increased apparent phosphorus digestibility and improved calcium and phosphorus metabolism in weaned piglets. This combined supplementation also enhanced calcium content, bone mineral density, and overall bone quality of the metacarpal and rib bones. Additionally, supplementation with 25-OH-VD_3_, either alone or in combination with phytase and probiotics, increased the abundance of beneficial gut bacteria, including Eggerthellaceae, Eubacterium, and Lachnospiraceae. However, in low-calcium and low-phosphorus diets, the combined inclusion of 25-OH-VD_3_, phytase, and probiotics did not significantly affect growth performance, mineral metabolism, or bone development in weaned piglets.

## Figures and Tables

**Table 1:** The ingredients and nutrient composition of diets at analyzed level at (DM basis, %).

Items	Treatments				
	CON	LCP	LH	LC	LHC
Ingredients					
Corn	56.82	56.49	56.49	56.49	56.49
Soybean meal	13.50	13.18	13.18	13.18	13.18
Extruded full-fat soybean	13.06	13.05	13.05	13.05	13.05
Fish meal	4.50	4.50	4.50	4.50	4.50
Whey powder	3.00	3.00	3.00	3.00	3.00
Wheat shorts	5.64	6.72	6.72	6.72	6.72
L-Lys HCl (78%)	0.58	0.58	0.58	0.58	0.58
DL-Met (99%)	0.07	0.07	0.07	0.07	0.07
L-Thr (98.5%)	0.14	0.15	0.15	0.15	0.15
L-Trp	0.03	0.02	0.02	0.02	0.02
CaHPO_4_·2H_2_O	0.64	0.34	0.34	0.34	0.34
CaCO_3_	0.62	0.50	0.50	0.50	0.50
NaCl	0.30	0.30	0.30	0.30	0.30
Mold inhibitor	0.10	0.10	0.10	0.10	0.10
Premix ^1^	1.00	1.00	1.00	1.00	1.00
Chemical composition					
ME (MJ/kg) ^2^	13.9	13.9	13.9	13.9	13.9
CP ^3^	20.10	20.10	20.10	20.10	20.10
Calcium ^3^	0.75	0.62	0.62	0.62	0.62
Phosphorus ^3^	0.35	0.29	0.29	0.29	0.29

^1^ Premix provides the following per kg diet: Cu (as copper sulfate), 100 mg; Fe (as ferrous sulfate), 100 mg; Zn (as zinc oxide), 120 mg; Mn (as manganese sulfate), 20 mg; I (as calcium iodate), 0.3 mg; and Se (as sodium selenite), 0.3 mg; vitamin A, 3, 800 IU; 25-OH-D_3_, 50 µg/kg; vitamin E, 10 IU; vitamin K, 1 mg; choline, 200 mg; pantothenic, 5 mg; vitamin B2, 2 mg; folic acid, 0.8 mg; vitamin B1, 1 mg; vitamin B6, 1 mg; biotin, 0.08 mg; vitamin B12, 0.01 mg; 0.3% titanium dioxide. ^2^ Calculated values. ^3^ Analyzed values. Abbreviations: CON = basal diet + 50 µg/kg 25-OH-VD_3_; LCP = basal diet with 17% reduced calcium and phosphorus + 50 µg/kg 25-OH-VD_3_; LH = basal diet with 17% reduced calcium and phosphorus + 50 mg/kg phytase + 50 µg/kg 25-OH-VD_3_; LC = basal diet with 17% reduced calcium and phosphorus + 10 mg/kg probiotics + 50 µg/kg 25-OH-VD_3_; LHC = basal diet with 17% reduced calcium and phosphorus + 50 mg/kg phytase + 10 mg/kg probiotics + 50 µg/kg 25-OH-VD_3_. Same as the tables and figures below.

**Table 2:** Growth performance and apparent nutrient digestibility of weaned piglets fed low Ca and P diets supplemented with 25-OH-VD_3_ combined with phytase, and probiotics.

Items	Treatments					SEM	*p*-Value
	CON	LCP	LH	LC	LHC		
Growth performance							
Initial weight (kg)	7.19	7.23	7.21	7.18	7.24	0.175	1.000
Final weight (kg)	16.46	17.08	16.81	16.57	17.16	0.358	0.966
BWG (kg)	9.27	9.85	9.61	9.39	9.93	0.254	0.912
ADG (g)	331.1	351.9	343.1	335.2	354.6	9.081	0.912
ADFI (g/d)	591.4	587.0	587.9	569.2	573.9	0.014	0.984
F/G	1.78	1.67	1.71	1.69	1.62	0.023	0.201
Apparent nutrient digestibility							
DM, %	87.5	87.6	87.6	87.5	87.6	0.04	0.672
CP, %	82.6 ^a^	81.0 ^ab^	81.6 ^a^	79.5 ^b^	79.5 ^b^	0.34	0.007
Calcium, %	69.7	67.4	70.4	67.9	67.7	1.72	0.979
Phosphorus, %	52.8 ^c^	58.3 ^bc^	68.1 ^a^	58.9 ^bc^	65.3 ^ab^	1.50	0.005

^a, b, c^ Different superscripts within a row indicate significant differences (*p* < 0.05). Data are presented as means (*n* = 7). Abbreviations: BWG = body weight gain; ADG = average daily gain; ADFI = average daily feed intake; F/G = feed to gain ratio.

**Table 3:** Effects of 25-OH-VD_3_ combined with phytase and probiotics with low Ca and P level diet on organ index of weaned piglets.

Items	Treatments					SEM	*p*-Value
	CON	LCP	LH	LC	LHC		
Organ index, g/kg							
Heart	5.16	5.60	5.24	5.59	5.06	0.211	0.953
Liver	27.79	30.31	32.59	34.18	34.24	0.953	0.168
Spleen	2.41	2.26	2.30	2.87	2.27	0.143	0.652
Lung	14.13	13.73	13.65	14.57	12.83	0.539	0.900
kidney	5.10	5.07	5.31	5.03	5.38	0.116	0.841
Pancreas	1.80	2.51	2.31	2.69	2.25	0.120	0.176
Stomach	8.91	8.47	8.90	8.84	8.93	0.267	0.176

Data are presented as means (*n* = 7). Organ index: Organ weight (g)/Body weight (kg).

**Table 4:** Effects of 25-OH-VD_3_ combined with phytase and probiotics with low Ca and P level diet on the serum biochemical indices of weaned piglets.

Items	Treatments					SEM	*p*-Value
	CON	LCP	LH	LC	LHC		
Albumin, g/L	41.1	29.5	32.3	34.6	38.6	1.55	0.116
Glucose, mmol/L	9.77 ^a^	7.22 ^b^	8.06 ^ab^	7.10 ^b^	7.11 ^b^	0.327	0.030
Blood urea nitrogen, mmol/L	2.74 ^a^	2.65 ^a^	1.54 ^ab^	1.87 ^ab^	1.20 ^b^	0.196	0.038
Total cholesterol, g/L	2.02 ^c^	3.44 ^a^	2.44 ^bc^	2.59 ^b^	2.26 ^bc^	0.108	0.001
Calcium, mmol/L	2.53	2.57	2.50	2.51	2.50	0.027	0.919
Phosphorus, mmol/L	3.19	3.28	3.44	2.40	3.31	0.128	0.066
1,25-(OH)_2_-D_3_, ng/L	831	744	691	760	728	23.4	0.437
25-OH-VD_3_, μg/L	14.0	14.1	12.6	14.7	12.1	0.51	0.481
Anti-tartrate acid phosphatase, (ug/L)	7.63	7.19	7.38	6.00	7.03	0.358	0.680
Bone Alkaline Phosphatase, ug/L	7.26 ^a^	6.30 ^a^	7.14 ^a^	5.10 ^a^	10.93 ^b^	0.620	0.028
Parathyroid hormone, ng/L	17.0 ^bc^	29.0 ^a^	15.1 ^c^	23.3 ^ab^	18.8 ^bc^	1.35	0.003
Calcitonin, ng/L	15.4	15.5	15.4	15.6	15.7	0.07	0.320

^a, b, c^ Different superscripts within a row indicate significant differences (*p* < 0.05). Data are presented as means (*n* = 7).

**Table 5:** Effects of 25-OH-VD_3_ combined with phytase and probiotics on the bone performance of weaned piglets with low calcium and phosphorus levels (*n* = 7).

Items	Treatments					SEM	*p*-Values
	CON	LCP	LH	LC	LHC		
Metacarpal Bone							
BMD, g/mL	0.42 ^b^	0.49 ^b^	0.46 ^b^	0.42 ^b^	0.57 ^a^	0.042	<0.001
Skimmed bone weight, g	1.79	1.62	1.62	1.35	1.89	0.057	0.408
Calcium, %	20.1	17.5	17.1	17.9	18.7	0.52	0.386
Phosphorus, %	5.71	5.47	5.41	5.63	5.86	0.087	0.529
Ribs bone							
BMD, g/mL	0.58 ^b^	0.56 ^b^	0.56 ^b^	0.57 ^b^	0.73 ^a^	0.021	0.029
Skimmed bone weight, g	1.11 ^b^	1.07 ^b^	1.13 ^b^	0.98 ^b^	1.33 ^a^	0.034	0.012
Calcium, %	18.9	19.83	18.8	19.1	21.1	0.32	0.126
Phosphorus, %	6.42	6.06	5.88	6.02	6.05	0.146	0.839

^a, b^ Different superscripts within a row indicate significant differences (*p* < 0.05). Data are presented as means (*n* = 7). Abbreviations: BMD, bone mineral density.

**Table 6:** Effects of 25-OH-VD_3_ combined with phytase and probiotics on the jejunal mucosal morphology of weaned piglets.

Items	Treatments					SEM	*p*-Value
	CON	LCP	LH	LC	LHC		
Villus height, μm	532 ^a^	362 ^c^	465 ^b^	494 ^ab^	510 ^ab^	10.4	0.001
Crypt depth, μm	167 ^a^	141 ^b^	144 ^b^	127 ^b^	147 ^ab^	3.38	0.004
V/C	3.30 ^b^	2.72 ^c^	3.33 ^b^	4.01 ^a^	3.59 ^ab^	0.08	0.001

^a, b, c^ Different superscripts within a row indicate significant differences (*p* < 0.05). Data are presented as means (*n* = 7).

## Data Availability

The raw data supporting the conclusions of this article will be made available by the authors on request.

## Supplementary Materials

The following supporting information can be downloaded at: https://www.mdpi.com/article/10.3390/ani16020278/s1, Table S1: The sequences of primer.

## Abbreviations

The following abbreviations are used in this manuscript:

ADFI	Average feed intake
ADG	Average daily weight gain
ASV	Amplicon sequence variant
CaBP-D9k	Calcium-binding protein D9k.
CaSR	Calcium sensing receptor
CYP27B1	Cytochrome P450 27B1
CP	Crude protein
DM	Dry matter
F/G	Feed to gain ratio
LDA	Linear discriminant analysis
SLC34A1	Solute carrier family 34 (type II sodium/phosphate transporter), member 1
TRPV5	Transient receptor potential cation channel subfamily V member 5
VDR	Vitamin D receptor
25-OH-VD_3_	25-hydroxyvitamin D_3_
