# Supplementary material for: Synergistic Effects of 25-Hydroxyvitamin D3, Phytase, and Probiotics on Growth, Calcium and Phosphorus Metabolism, and Bone Development in Weaned Piglets Fed Low Ca-P Diets
Source: Animals (Basel). 2026 Jan 16;16(2):278. doi: 10.3390/ani16020278 (PMC12837494; doi:10.3390/ani16020278)
Supplement: Supplementary file 1 [file animals-16-00278-s001.zip › animals-4050600-supplementary.pdf]

Table S1. The sequences of primer.

Gene	Primer sequence (5′→3′)	Length of PCR (bp)	Tm value (ºC)
β-actin	F: TGCGGCATCCACCAAACTA<br>R: CGTAGAGGTCCTTGCGGATGT	70	60
SLC34A1	F: CTATCTCCCCGCTCCCAGT<br>R: GAAGGCATAGGCTGAGGTCC	113	60
SLC34A2	F: TCGTGTGCTCCTTGGATGTG<br>R: CAACACGGAGAGCCAGTTGA	356	60
SLC34A3	F: GCCCCTCGCACTCACAC<br>R: CTTCCCACCCGCTCTTG	269	60
TRPV5	F: GCTCCCTTGAACCACATCCCT<br>R: GTGGGCTTCATCCAGTTGCT	162	60
TRPV6	F: GCTGTGGTCATCCTGGGCTTTG<br>R: AATGATGGCGAAGGCGGCATAG	204	60
CYP27B1	F: ACACAGAGACCTTCATCCGC<br>R: GTGTCCACTCCAGCCAGTAG	313	60
VDR	F: TGGTTGGAAGTGTCTGGGAG<br>R: GGGGTCAGGTAAGGAAGTGC	117	60
CaSR	F: ACTGAGCCCTTTGGGATTG<br>R: TTGACGATGGGCGTGTTT	107	60
CaBP-D9k	F: AGAGCAAATGCACCTCTTGG<br>R: CATGTGAGCGCATAGAAGGA	105	60
CaBP-D28k	F: TATGCAGCCAAAGAAGGGGAT<br>R: CTAGGGTTCTCGGACCTTTCAG	103	60

Abbreviations: SLC34A1/ SLC34A2/ SLC34A3, solute carrier family 34 (type II sodium/phosphate transporter), member 1/ member 2/ member 3; TRPV5/ TRPV 6, transient receptor potential cation channel subfamily V member 5/ member 6; CYP27B1, cytochrome P450 27B1; VDR, vitamin D receptor; CaSR, calcium sensing receptor; CaBP-D9k, calcium-binding protein D9k; CaBP-D28k, calcium-binding protein D28k. All genes were normalized using the expression level of β-actin.
